# Efficacy and safety of concomitant catheter ablation and left atrial appendage closure compared to catheter ablation only – a meta-analysis

**DOI:** 10.1007/s10840-026-02418-3

**Published:** 2026-08-17

**Authors:** Isabel Rattka, Christine Poch, Tabea Deichsel, Eimo Martens, Karl-Ludwig Laugwitz, Alexander Steger, Manuel Rattka

**Affiliations:** School of Medicine and Health, Department of Clinical Medicine—Clinical Department of Cardiology, University Medical Centre, Technical University of Munich, Ismaninger Straße 22, Munich, 81675 Germany

**Keywords:** Atrial fibrillation, Left atrial appendage closure, Catheter ablation, Pulmonary vein isolation, Arrhythmia recurrence, Meta-analysis

## Abstract

**Background:**

Atrial fibrillation (AF) carries a substantial ischemic stroke risk originating predominantly from the left atrial appendage (LAA). Catheter ablation (CA) is the most effective rhythm-control strategy, while percutaneous LAA closure (LAAC) provides mechanical stroke prophylaxis. Combining both in a single one-stop procedure is increasingly performed, yet data on its rhythmic and periprocedural consequences remain scarce.

**Methods:**

We performed a systematic review and meta-analysis to compare the efficacy and safety of combined CA + LAAC versus CA-only in patients with AF. PubMed, Scopus and Web of Science were searched through June 12th 2026. The primary efficacy and safety outcomes were arrhythmia recurrence and periprocedural pericardial effusion requiring drainage, respectively. Binary endpoints were pooled as odds ratios (OR), continuous endpoints as mean differences (MD), using a random-effects model with heterogeneity reported as I^2^.

**Results:**

Fourteen studies comprising 3,274 patients (1,505 CA + LAAC; 1,769 CA-only) were included. Arrhythmia recurrence was significantly more frequent after combined CA + LAAC (OR = 1.56, 95% CI = 1.11–2.19, I^2^ = 47%). Systemic thromboembolism was numerically lower but did not differ significantly (OR = 0.66, 95% CI = 0.37–1.21, I^2^ = 0%). Total procedure time was significantly longer for the combined procedure (MD = + 9.34 min, 95% CI = 0.63–18.05, I^2^ = 90%). Periprocedural pericardial effusion requiring drainage did not differ between groups (OR = 1.19, 95% CI = 0.59–2.44, I^2^ = 0%).

**Conclusion:**

Combined CA + LAAC was associated with significantly higher arrhythmia recurrence than CA-only, potentially attributable to limited substrate modification in the one-stop setting. The primary safety outcome, pericardial effusion requiring drainage, did not differ between groups. Randomised controlled trials are warranted to confirm these findings.

**Supplementary Information:**

The online version contains supplementary material available at https://doi.org/10.1007/s10840-026-02418-3.

## Introduction

Atrial fibrillation (AF) confers a substantially increased risk of ischemic stroke and systemic thromboembolism, the majority of which originates from the left atrial appendage (LAA) in non-valvular AF [1, 2]. Catheter ablation (CA), centred on pulmonary vein isolation (PVI), has been established as the most effective rhythm-control strategy in symptomatic AF, reducing arrhythmia burden and improving quality of life [1]. Restoration of sinus rhythm, however, does not abolish thromboembolic risk, and long-term oral anticoagulation (OAC) therefore remains recommended in patients at elevated stroke risk regardless of rhythm status [1, 3, 4]. Because continued OAC carries a bleeding risk and is often discontinued, percutaneous LAA closure (LAAC) has emerged as a mechanical alternative. Randomized trials have shown it to be non-inferior to anticoagulation for stroke prevention, although the strength of this evidence depends on the population studied, with a recent trial in patients at high risk of both stroke and bleeding failing to meet noninferiority [3–6]. In patients undergoing AF ablation specifically, LAAC has been shown to be non-inferior to anticoagulation for stroke prevention, with a lower risk of non-procedure-related bleeding [5]. Since CA addresses arrhythmogenesis and LAAC addresses appendage-borne thromboembolism, combining these mechanistically complementary interventions in a single "one-stop" procedure that shares femoral access and a transseptal puncture has gained increasing attention [7]. The rhythmic consequences of adding LAAC are mechanistically uncertain. The LAA is an established non-pulmonary-vein trigger implicated in arrhythmia recurrence, and electrical isolation of the appendage could therefore be protective. The mechanical occluder itself, however, may instead promote recurrence through local irritation [8, 9]. The current literature suggests that the combined approach is feasible, yet questions remain regarding its effect on rhythm outcomes, its thromboembolic protection relative to continued anticoagulation, and its periprocedural safety. Pericardial effusion in particular has emerged as a complication signal of the combined approach in prior analyses, plausibly reflecting the greater degree of left atrial instrumentation [10]. Since comparative data remain scarce, we performed a systematic review and meta-analysis comparing the efficacy and safety of combined CA and LAAC versus CA-only in patients with AF, focusing on arrhythmia recurrence, systemic thromboembolism, and periprocedural pericardial effusion.

## Methods

### Data sources and study selection

**Fig. 1:**
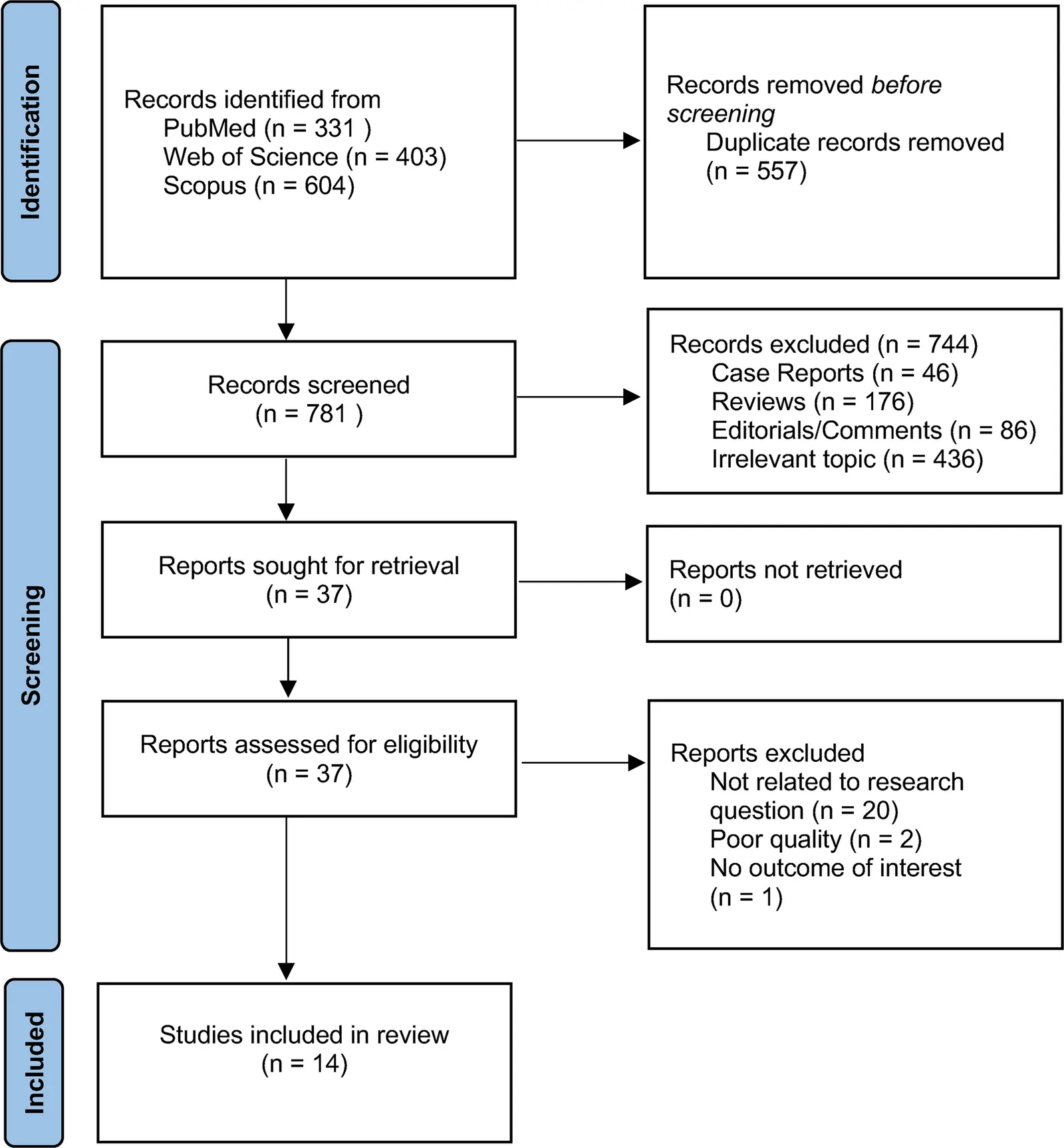
PRISMA flow diagram. Out of 1338 identified studies and after application of the inclusions and exclusion criteria fourteen studies were included in the quantitative synthesis

A comprehensive literature search was performed through PubMed, Scopus and Web of Science through June 12th, 2026 for studies comparing outcomes of AF patients treated by either CA + LAAC or CA-only using the keywords "left atrial appendage closure" and "pulmonary vein isolation" and "atrial fibrillation". The complete search strategy is shown in the supplements. We searched for randomized controlled studies, observational studies, and register- or database-based studies, without language restrictions. Conference abstracts, case reports, review articles, and posters were excluded. The flowchart illustrating the literature search strategy is shown in Fig. 1.

### Data extraction and study quality

Study selection, data extraction and quality assessment were performed as described in our previous meta-analyses [11, 12]. In short, two investigators independently reviewed all the articles, selected eligible studies and extracted relevant data. In case of any discrepancies, a third reviewer made the final decision. Data extraction used standardized extraction forms including information on authors, country of origin, publication year, study design, ablation energy source, type of LAAC device, method of confounding control, number of patients per group, number of complications at the time of the procedure and during follow-up (any access site complication, pericardial effusion with drainage, transient ischemic attack and/or stroke, any reported arterial thromboembolism or death), procedural metrics (total procedure time and total fluoroscopy time), arrhythmia recurrence during follow-up, and baseline characteristics (age, gender, percentage of patients with paroxysmal AF, number of patients with a history of TIA or stroke, arterial hypertension and diabetes mellitus, HAS-BLED score, and CHA₂DS₂-VASc score). The study quality was assessed as detailed by the NIH Quality Assessment Tool and studies were rated as being of either "good", "fair" or "poor" quality [13]. Studies rated as being of "poor" quality were excluded from further analysis. As all analyses were based on previously published studies, neither patient consent nor ethical committee approval was required for our study. This meta-analysis has been pre-registered at PROSPERO [CRD420261403552].

### Outcomes

We sought to compare the efficacy and safety of CA + LAAC versus CA-only in patients with AF. The primary efficacy outcome was arrhythmia recurrence during follow-up. Secondary efficacy outcomes were systemic thromboembolism (ischemic stroke, TIA, or systemic arterial embolism) during follow-up, all-cause death during follow-up, total procedure time, and total fluoroscopy time. The primary safety outcome was periprocedural pericardial effusion requiring drainage. Secondary safety outcomes were periprocedural groin complications and periprocedural systemic thromboembolism.

### Statistical analysis

The statistical methodology applied in this meta-analysis followed the approach used in our previous meta-analyses [11, 12]. In brief, data were collected in a Microsoft Excel® spreadsheet and outcome measures were calculated. If in the included studies continuous variables were reported only as median values with interquartile ranges, means and standard deviations were estimated by making use of the Box-Cox method, where feasible [14]. The random effects model was used to combine the estimates from the different studies. This was preferred over the fixed effects model since the random effects model gives a more conservative estimate. For binary endpoints the Mantel–Haenszel method was used, and the odds ratio (OR) including its 95% confidence interval is reported as the effect measure. For continuous endpoints the inverse-variance method was used, and the mean difference (MD) including its 95% confidence interval is reported. Between-study variance was estimated using the DerSimonian and Laird method, and confidence intervals were calculated using the Wald-type method. For the assessment of heterogeneity among studies, the statistical characteristic I^2^ is reported. Forest plots are used for graphical representation of the results, including all individual study results separately as well as the overall pooled result. Additionally, funnel plots were created to evaluate the risk of publication bias. Meta-analyses were performed using Review Manager (RevMan) version 5.4 (The Cochrane Collaboration, Copenhagen, Denmark). All tests were two-tailed, and results with a p-value of < 0.05 were considered statistically significant. The Preferred Reporting Items for Systematic Reviews and Meta-Analyses (PRISMA) statement was applied in this study [15].

Baseline characteristics were extracted separately for each treatment arm of every included study. Continuous variables were recorded as mean and standard deviation, and categorical variables as counts and percentages. To summarise the baseline profile across the entire included cohort, we accounted for the marked imbalance in sample size between studies, as a single administrative database contributed the majority of enrolled patients. Patient-weighted pooling would therefore have been disproportionately determined by this cohort and would not have reflected the typical study population. Accordingly, patient age and the prevalence of categorical comorbidities were summarised at the study level as the median and range across studies, giving each study equal weight. The CHA₂DS₂-VASc and HAS-BLED scores, which were not reported in the largest administrative cohort, were additionally pooled across the reporting studies as sample-size-weighted means, with a corresponding pooled standard deviation derived by combining the within-study and between-study variance. These summaries are descriptive only; no formal statistical comparison of baseline characteristics between treatment groups was performed.

## Results

### Literature search, quality assessment and baseline characteristics

Based on our search strategy, 1338 studies were identified from the PubMed, Scopus and Web of Science databases. After removal of 557 duplicates, 781 records were screened, of which 744 were excluded (46 case reports, 176 reviews, 86 editorials/comments, and 436 records on an irrelevant topic), leaving 37 articles assessed for eligibility. Finally, after exclusion of another 20 articles not related to the research question, 2 studies of poor quality as per the NIH tool, and 1 study that reported no outcome of interest, 14 studies were included in the quantitative synthesis (Fig. 1) [7, 9, 16–27]. According to the NIH Study Quality Assessment tool, 5 studies were rated as being of "good" and 9 studies of "fair" methodological quality, (Table 1). Two of the included studies were or derived from randomized controlled trials [7, 9] whereas the remaining 12 studies were retrospective observational cohorts or registry-based analyses.

**Table 1:** Summary of included studies

Study	Country/Database	mono-/multicentric	Study design	NIH	No. of patients	Type of Ablation	LAAC device	Confounding control
Wang et al. 2022	China	monocentric	retrospective	fair	80	RFA	N/A	none
Yang et al. 2021	China	monocentric	retrospective	fair	130	RFA	Watchman	PS-matched
Pasupula et al. 2023	NRD DB	multicentric	retrospective	fair	781	RFA/CBA	N/A	PS-matched
Romanov et al. 2015	Russia	monocentric	RCT	good	89	RFA	Watchman	RCT
Zhu et al. 2020	China	monocentric	retrospective	fair	112	RFA	mixed	PS-matched
Pelissero et al. 2017	Italy	monocentric	retrospective	fair	42	RFA/CBA	mixed	none
Mo et al. 2020	China	monocentric	retrospective	good	152	RFA	Watchman	PS-matched
Yang et al. 2022	China	monocentric	retrospective	good	124	RFA	Watchman	Matched for age&sex
Ding et al. 2024	China	monocentric	retrospective	fair	214	RFA/CBA	Watchman	PS-matched
Tam et al. 2025	China	multicentric	retrospective	fair	88	PFA	Watchman	none
Ren et al. 2020	China	monocentric	retrospective	fair	304	CBA	mixed	none
Lv et al. 2024	China	monocentric	retrospective	fair	178	RFA	Watchman	PS-matched
Yu et al. 2026	China	monocentric	retrospective	good	326	RFA	Watchman	PS-matched
Saliba et al. 2025	worldwide	multicentric	RCT	good	654	mixed	Watchman	RCT

*DB* database, *NRD* nationwide readmission database, *NIH* study quality as per *NIH* quality assessment tool, *LAAC* left atrial appendage closure, *RCT* randomized controlled trial, *RFA* radiofrequency ablation, *CBA* cryoballoon ablation, *PFA* pulsed field ablation, *PS* propensity score

A total of 3,274 patients were enrolled across the included studies, comprising 1,769 patients undergoing CA-only and 1,505 patients undergoing combined CA + LAAC. Across studies, the median patient age was 66 years (range 60 to 74) and the median proportion of female patients was 44% (range 25% to 51%). The included population was clinically heterogeneous with respect to arrhythmia type: the median proportion of patients with paroxysmal AF was 50% and ranged from 0 to 100% between studies, reflecting the inclusion of both predominantly paroxysmal and predominantly persistent cohorts. The median prevalence of arterial hypertension was 70% (range 56% to 82%), of diabetes mellitus 22% (range 13% to 31%), and of prior stroke or transient ischemic attack 26% (range 10% to 54%). The pooled mean CHA₂DS₂-VASc score was 3.5 ± 1.5 and the pooled mean HAS-BLED score 2.2 ± 1.3. Baseline characteristics were broadly comparable between the two treatment groups, with the combined CA + LAAC group being marginally older (68.8 vs 68.1 years) and showing a slightly higher mean CHA₂DS₂-VASc score (3.6 vs 3.3). Detailed baseline characteristics and further study characteristics are listed in Table 2 and Supplementary Table 1.

**Table 2:** Summary of patient characteristics

Study	Group	No. patients	Age	Female	paroxAF	AHT	DM	Stroke/TIA	CHA_2_DS_2_-VASC	HAS-BLED
Wang et al. 2022	PVI	40	62.6 ± 5.3	50	N/A	67.5	30.0	20.0*	N/A	N/A
	PVI + LAAC	40	63.6 ± 4.5	52.5	N/A	67.5	32.5	17.5*	N/A	N/A
Yang et al. 2021	PVI	65	60.7 ± 9.1	38	0 (persAF)	77	15	43	4.0 ± 1.5	N/A
	PVI + LAAC	65	61.8 ± 7.9	34	0 (persAF)	69	14	52	3.0 ± 1.5	N/A
Pasupula et al. 2023	PVI	406	74.0 ± 9.2	45.9	53.1	81.1	24.6	N/A	N/A	N/A
	PVI + LAAC	375	74.0 ± 9.2	46.6	49.1	83.2	29.6	N/A	3.8 ± 1.3	N/A
Romanov et al. 2015	PVI	44	60 ± 6	40.9	56.8	75.0	25.0	13.6	2.3 ± 0.7	3.4 ± 0.8
	PVI + LAAC	45	60 ± 5	37.8	53.3	84.4	20.0	8.9	2.2 ± 0.6	3.5 ± 0.8
Zhu et al. 2020	PVI	56	64.8 ± 8.5	30.3	53.6	69.6	19.6	32.1	4.1 ± 1.7	1.8 ± 1.1
	PVI + LAAC	56	65.2 ± 6.6	41.1	42.9	69.6	23.2	30.4	4.3 ± 1.8	2.0 ± 1.3
Pelissero et al. 2017	PVI	21	68.4 ± 10.6	28.6	14.3	71.4	N/A	N/A	2.0 ± 0.9	3.1 ± 1.0
	PVI + LAAC	21	66.9 ± 10.4	33.3	19.0	66.7	N/A	9.5	2.8 ± 1.2	3.2 ± 0.8
Mo et al. 2020	PVI	76	69.5 ± 7.8	52.6	50.0	71.1	19.7	19.7	3.4 ± 1.4	2.6 ± 0.9
	PVI + LAAC	76	69.9 ± 7.9	48.8	48.7	73.7	18.4	30.3	3.6 ± 1.3	3.3 ± 1.1
Yang et al. 2022	PVI	62	62.5 ± 7.2	40.3	N/A	62.9	14.5	41.9	3.3 ± 1.7	N/A
	PVI + LAAC	62	64.2 ± 8.3	48.4	N/A	50.0	14.5	45.2	3.8 ± 1.6	N/A
Ding et al. 2024	PVI	107	66.3 ± 9.9	43.9	32.7	N/A	N/A	N/A	3.2 ± 1.1	2.8 ± 0.7
	PVI + LAAC	107	67.0 ± 8.7	47.7	28.0	N/A	N/A	N/A	3.3 ± 1.2	2.8 ± 0.8
Tam et al. 2025	PVI	52	56.2 ± 11.6	19.2	82.7	N/A	N/A	N/A	1.2 ± 1.3	N/A
	PVI + LAAC	36	65.7 ± 7.8	33.3	69.4	N/A	N/A	N/A	2.4 ± 1.2	N/A
Ren et al. 2020	PVI	262	66.3 ± 9.5	45.8	100	58.8	11.5	N/A	2.8 ± 1.9	2.7 ± 1.2
	PVI + LAAC	42	70.0 ± 7.6	38.1	100	61.9	19.1	N/A	3.8 ± 2.1	3.7 ± 1.2
Lv et al. 2024	PVI	89	65.6 ± 8.2	51.0	100	76	32	52	4.2 ± 1.7	3.1 ± 0.7
	PVI + LAAC	89	64.2 ± 7.5	42.0	100	70	27	56	4.2 ± 1.6	3.0 ± 1.0
Yu et al. 2026	PVI	163	68.1 ± 10.0	45.4	25.2	61.3	22.1	23.9	4.0 ± 1.5	N/A
	PVI + LAAC	163	67.6 ± 7.2	40.5	27.6	66.3	20.9	31.9	4.0 ± 1.5	N/A
Saliba et al. 2025	PVI	326	70.0 ± 7.3	31.9	56.7	N/A	N/A	11.7	3.5 ± 1.2	1.2 ± 0.8
	PVI + LAAC	328	70.1 ± 7.6	36.0	51.2	N/A	N/A	8.8	3.4 ± 1.2	1.1 ± 0.8

*LAAC* left atrial appendage closure, *PVI*, pulmonary vein isolation, *paroxAF* paroxysmal atrial fibrillation, *persAF* persistent atrial fibrillation, *AHT* arterial hypertension, *DM* diabetes mellitus; *TIA* transient ischemic attack, *TIA-only reported

### Efficacy outcomes

**Fig. 2:**
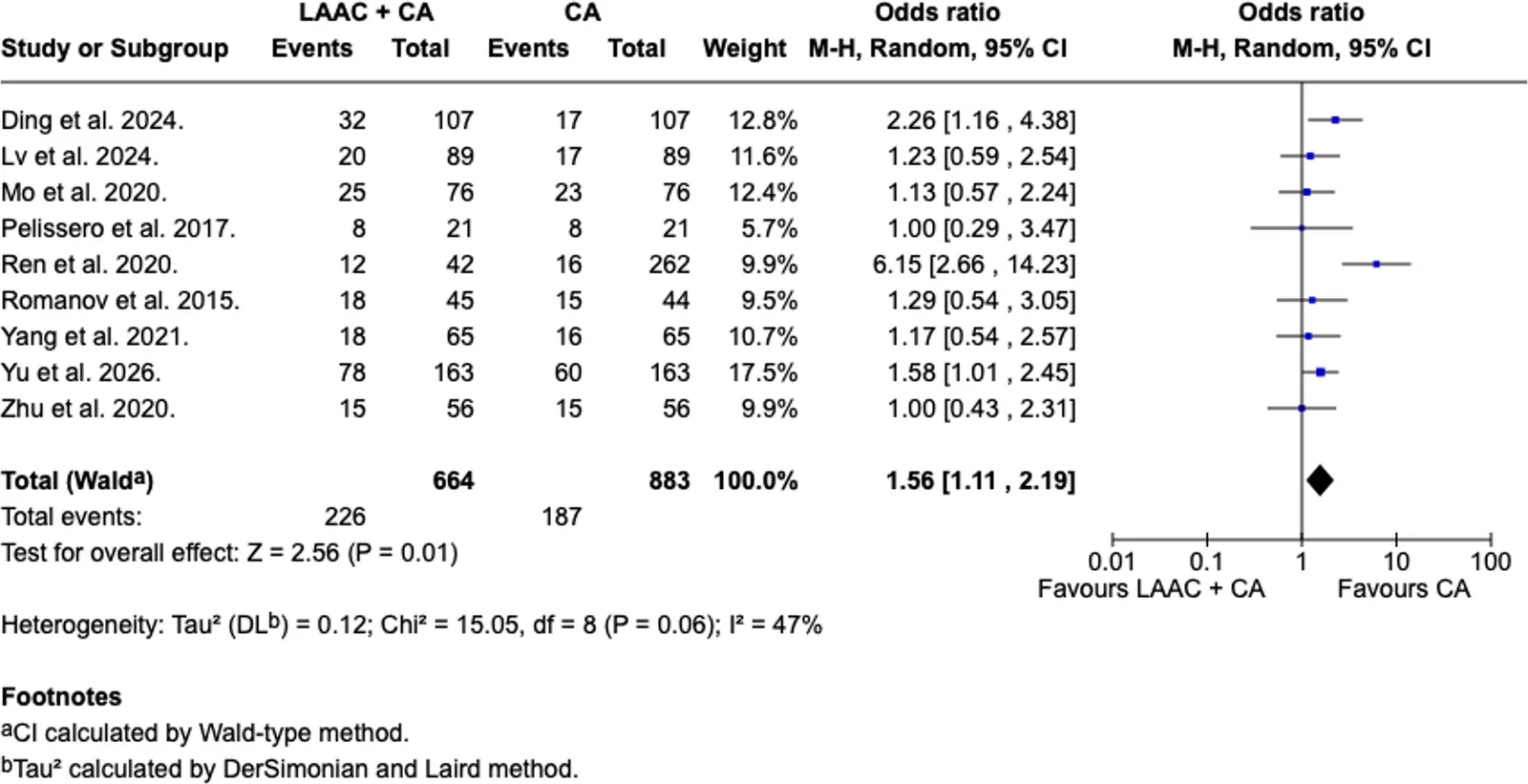
Forest plot – Arrhythmia recurrence. Arrhythmia recurrence was more frequent in patients undergoing concomitant LAAC + CA. LAAC, left atrial appendage closure; CA, catheter ablation; CI, confidence interval

The primary efficacy outcome, arrhythmia recurrence during follow-up, was reported in nine studies. Arrhythmia recurrence was significantly more frequent in patients undergoing combined CA + LAAC than in those undergoing CA-only (226/664 vs 187/883; OR = 1.56, 95% CI = 1.11–2.19, I^2^ = 47%; Fig. 2, Supplementary Fig. 1).

**Fig. 3:**
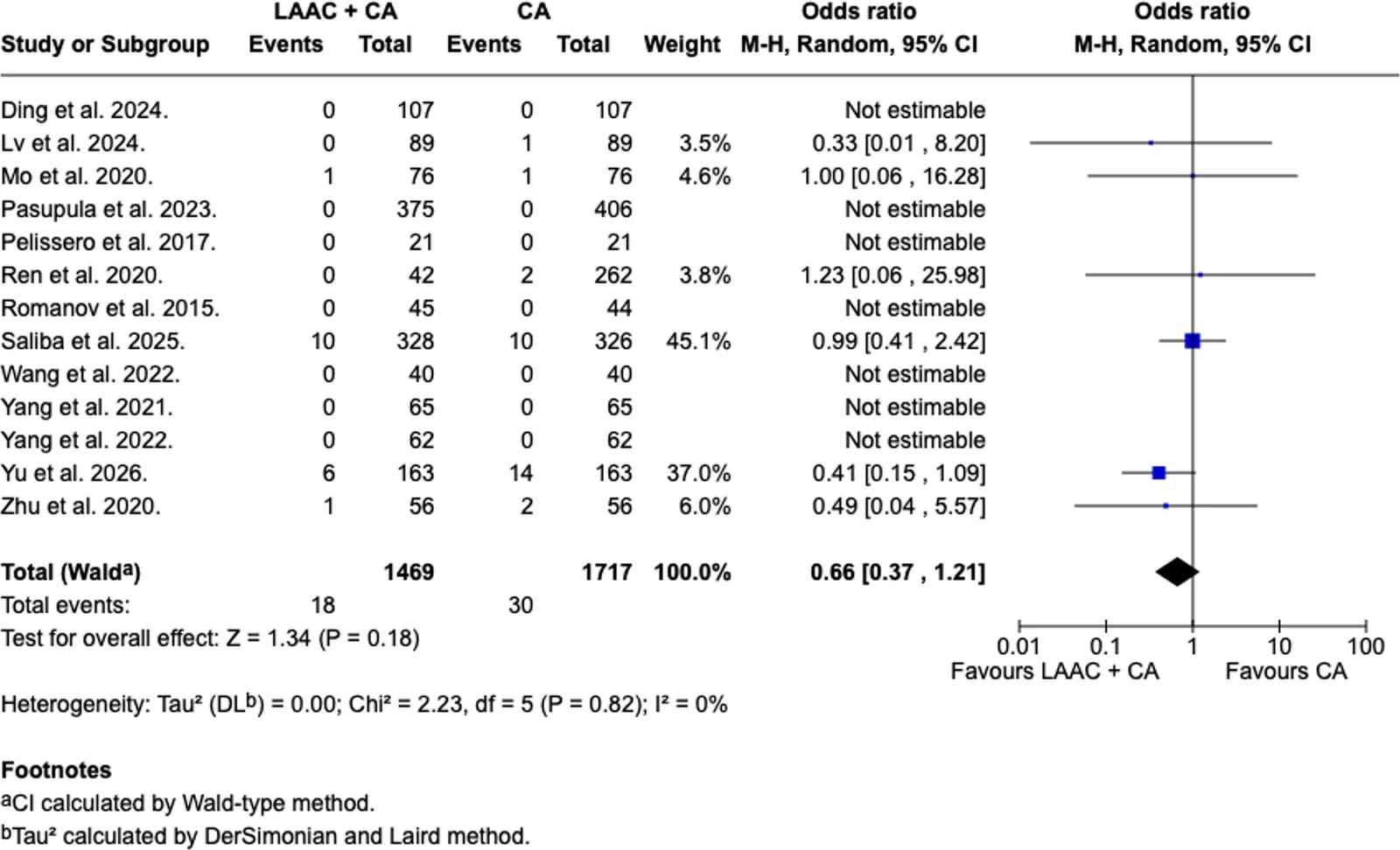
Forest plot – systemic thromboembolism. Overall, systemic thromboembolism did not differ between both groups. LAAC, left atrial appendage closure; CA, catheter ablation; CI, confidence interval

Systemic thromboembolism during follow-up, reported in 13 studies, did not differ significantly between groups, although a numerically lower event rate was observed in the combined cohort (18 vs 30 events; OR = 0.66, 95% CI = 0.37–1.21, I^2^ = 0%; Fig. 3, Supplementary Fig. 2). All-cause death during follow-up, available from eight studies, was comparable between the two groups (OR = 1.01, 95% CI = 0.60–1.70, I^2^ = 0%; Supplementary Figs. 3 and 4).

**Fig. 4:**
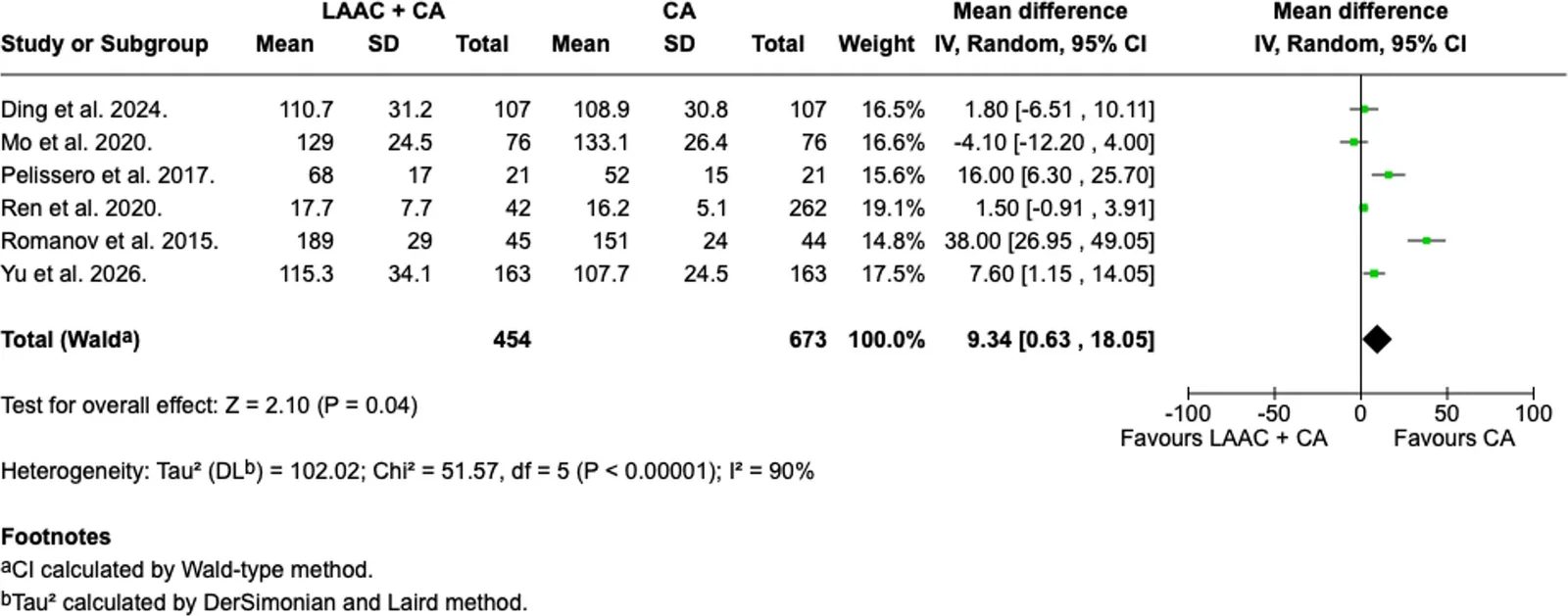
Forest plot – total procedure time. Total procedure time was lower in patients undergoing CA-only. LAAC, left atrial appendage closure; CA, catheter ablation; CI, confidence interval

Total procedure time, reported in six studies, was significantly longer for the combined procedure (mean difference [MD] = + 9.34 min, 95% CI = 0.63–18.05, I^2^ = 90%; Fig. 4, Supplementary Fig. 5). Total fluoroscopy time, available from three studies, did not differ significantly between groups (MD = + 2.81 min, 95% CI = − 0.88–6.51, I^2^ = 98%; Supplementary Figs. 6 and 7).

### Safety outcomes

**Fig. 5:**
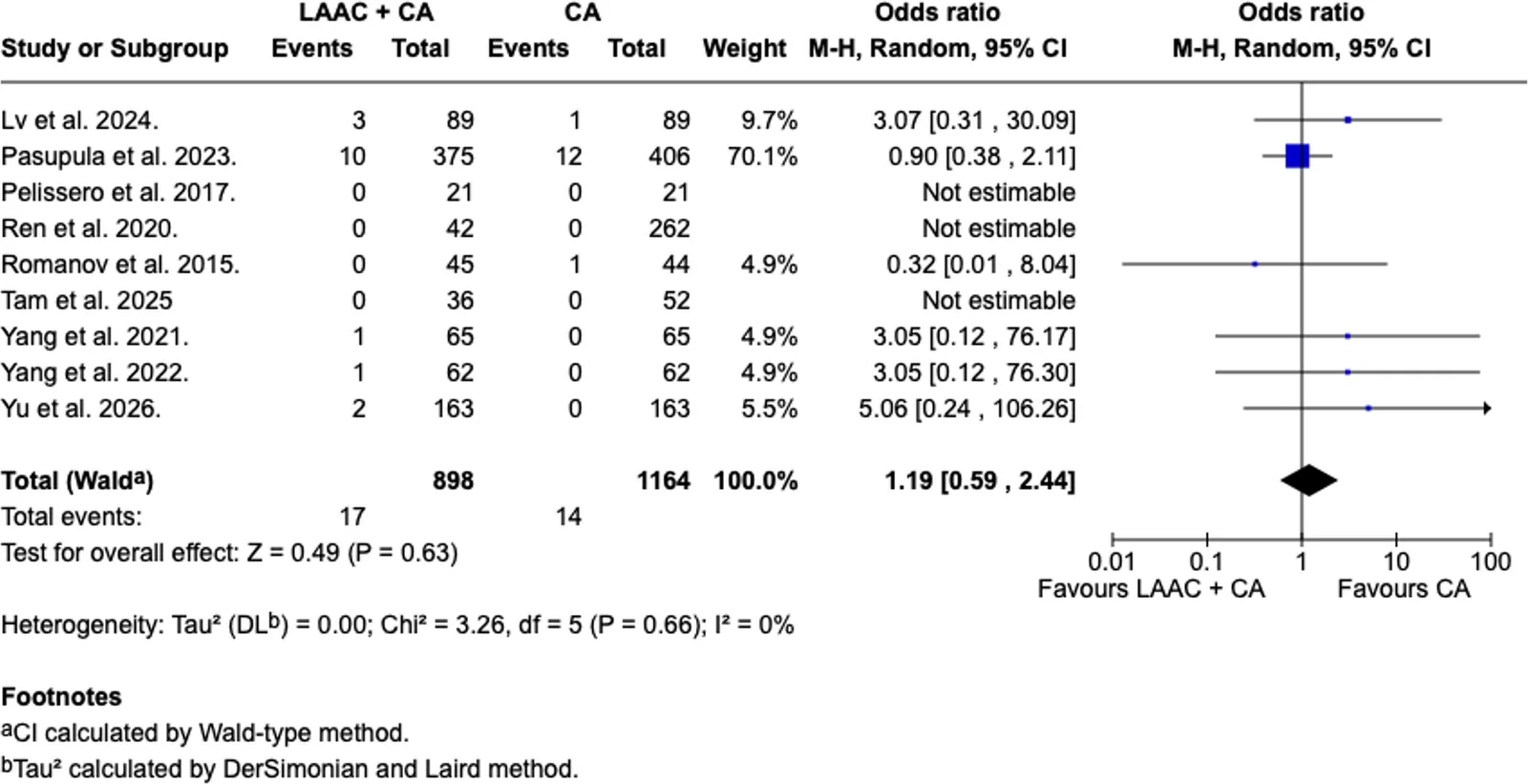
Forest plot – pericardial effusion requiring drainage. Pericardial effusion requiring drainage did not differ significantly between both groups. LAAC, left atrial appendage closure; CA, catheter ablation; CI, confidence interval

Pericardial effusion requiring drainage was assessed as the primary safety endpoint and was reported in nine studies. There was no significant difference between groups, with substantial between-study heterogeneity (17 vs 14 events; OR = 1.19, 95% CI = 0.59–2.44, I^2^ = 0%; Fig. 5, Supplementary Fig. 8).

Periprocedural groin complications, reported in five studies, did not differ significantly between groups (OR = 1.40, 95% CI = 0.43–4.58, I^2^ = 0%; Supplementary Figs. 9 and 10). Periprocedural systemic thromboembolism, available from four studies, showed no significant difference between groups (OR = 1.28, 95% CI = 0.22–7.57, I^2^ = 13%; Supplementary Figs. 11 and 12).

## Discussion

In this meta-analysis encompassing 14 studies and a total of 3,274 patients, we evaluated the efficacy and safety of combined CA + LAAC compared with CA-only in patients with atrial fibrillation. The principal findings are as follows: (1) arrhythmia recurrence during follow-up, the primary efficacy endpoint, was significantly more frequent after combined CA + LAAC than after CA-only. (2) Systemic thromboembolism during follow-up was numerically less frequent in the combined group, although this difference did not reach statistical significance. (3) With regard to safety, periprocedural pericardial effusion requiring drainage did not differ significantly between groups.

### Arrhythmia recurrence

Contrary to the assumption that concomitant appendage closure would be neutral with respect to rhythm outcomes, we observed a significantly higher rate of arrhythmia recurrence in the combined group. Several factors might contribute to this finding.

First, the available evidence is almost entirely observational, and confounding by indication is possible. Patients selected for concomitant LAAC frequently carry a higher thromboembolic and bleeding risk, a greater burden of persistent atrial fibrillation, more pronounced atrial cardiomyopathy and larger left atrial dimensions, all of which are independently associated with arrhythmia recurrence after ablation [1, 2, 26, 28, 29]. Reassuringly, most of the included cohorts applied propensity-score matching or multivariable adjustment, and two studies were randomized which limits the extent to which such imbalance can account for the observed signal. Nevertheless, residual confounding cannot be excluded. The pooled baseline profile of the present analysis still showed a marginally older combined group with a slightly higher mean CHA₂DS₂-VASc score, indicating that a modest risk gradient persisted even across this predominantly adjusted evidence base. The recurrence signal should therefore be regarded as hypothesis-generating rather than as evidence of a causal detrimental effect of appendage closure on rhythm control.

Second, a procedural mechanism may contribute. In a one-stop setting, operators may deliberately limit the extent of ablation to contain total procedure duration, periprocedural risk and cumulative left atrial dwell-time. Such under-ablation, for example restriction to pulmonary vein isolation without additional substrate modification, or avoidance of lesions close to the intended device landing zone, may leave residual arrhythmogenic substrate and predispose to recurrence [23, 26]. Consistent with this, deliberate correction of insufficient ablation has been shown to restore rhythm outcomes [23].

Third, the appendage and its interaction with the device may play a role. The appendage is a recognised source of non-pulmonary-vein triggers, and electric isolation can contribute to successful rhythm control. However, mechanical irritation of the peri-appendage tissue and the left lateral ridge by device manipulation might elevate arrhythmia burden, especially in the early post-procedural phase [9]. Nonetheless, these irritative effects appear to resolve over the following months and are therefore more likely to drive early than sustained recurrence [9].

Recent evidence helps discriminate between these mechanisms. In a large sub-analysis of the OPTION trial, AF recurrence after ablation did not differ significantly between patients randomized to LAAC or oral anticoagulation, and within the LAAC arm, recurrence was similar for concomitant and sequential device implantation despite a significantly higher burden of persistent atrial fibrillation in the concomitant group [30]. It therefore appears unlikely that sustained mechanical irritation of the peri-appendage tissue accounts for the higher rate of arrhythmia recurrence observed in the combined group in our analysis. Because ablation strategy in the OPTION trial was not assigned differentially between groups, this finding instead supports under-ablation as the more relevant factor underlying the result of the present meta-analysis [5]. When concomitant PVI and LAAC are indicated, procedural planning should therefore ensure that ablation strategy is not compromised, to avoid under-ablation and subsequent arrhythmia recurrence.

### Systemic thromboembolism

Systemic thromboembolism during follow-up occurred numerically less often in the combined group, although the difference did not reach statistical significance and the analysis rested on a small number of events. The direction of effect is mechanistically plausible. Mechanical occlusion of the appendage removes the predominant source of cardiogenic thrombi in non-valvular atrial fibrillation, while concomitant restoration of sinus rhythm is thought to attenuate the contribution of atrial cardiomyopathy and recurrent arrhythmia to thromboembolic risk [1, 2, 29]. The wide confidence interval, however, precludes any firm inference, and the scarcity of events itself warrants closer consideration. This scarcity is not unexpected and is best interpreted against the low baseline ischemic risk of the contemporary post-ablation population. In recent randomized trials of patients followed after successful ablation, the residual ischemic risk was very low and fell below the conventional threshold for anticoagulation, with neither continued anticoagulation-only nor an oral anticoagulant over aspirin reducing ischemic events [31, 32]. Against such a low event floor, any incremental thromboembolic benefit of adding mechanical appendage closure is necessarily small and difficult to demonstrate without very large sample sizes. The magnitude of any benefit therefore likely depends on baseline stroke risk: these low-event trials enrolled comparatively low-risk cohorts (mean CHA₂DS₂-VASc 2 to 2.2) [31, 32], whereas the OPTION trial that established closure as a valid alternative to anticoagulation after ablation enrolled a higher-risk population (mean CHA₂DS₂-VASc 3.5) matching the pooled profile of the present analysis [5, 7]. The thromboembolic advantage of combined CA and LAAC is thus likely concentrated in higher-risk patients, whereas in lower-risk patients the marginal benefit is negligible.

### Pericardial effusion requiring drainage

Combined CA + LAAC was not associated with a significantly different rate of pericardial effusion requiring drainage compared with CA alone. However, most of the studies showed a numerically higher event rate in the combined arm, albeit never reaching individual significance [17, 22, 25, 26]. This direction is biologically plausible. The combined procedure entails a longer left atrial dwell time, additional device manipulation and, depending on the chosen workflow, a second transseptal puncture, each of which may contribute to mechanical injury of atrial structures [10]. Two factors may nonetheless explain why the combined procedure does not show the higher effusion rate that might be expected. In the predominant workflow catheter ablation precedes appendage closure, so that an effusion arising during ablation prompts the operator to abort before device implantation and is attributed to the ablation phase, which tends to equalise rather than inflate the apparent periprocedural risk of the combined arm. Furthermore, the modest difference in total procedure time observed in the present analysis argues against a substantial additional mechanical burden. Thus, the current evidence can therefore neither confirm nor exclude a small true excess, and adequately powered studies using a standardised, intervention-based definition of pericardial effusion are warranted.

## Limitations

This meta-analysis has several limitations. Most included studies were retrospective, single-centre, and predominantly conducted in China, and not all used propensity-score matching. In unmatched cohorts the combined-procedure group was typically higher-risk at baseline, so residual confounding cannot be excluded, although this is attenuated by the predominance of adjusted cohorts. Procedural timing, follow-up duration, arrhythmia recurrence definitions, blanking period, and antiarrhythmic drug protocols were heterogeneous and inconsistently reported across studies, precluding a formal quantitative subgroup analysis. Blanking-period AF burden could not be separately extracted in most studies, so a possible contribution of transient device-related irritation to the observed recurrence signal could not be formally tested. Only one included study used pulsed field ablation, precluding subgroup analysis by ablation energy source, and future meta-analyses should revisit this question once sufficient data become available. Fluoroscopy time was reported by only three studies, limiting inference on the role of imaging guidance during device deployment. Several endpoints rested on few events and, consequently, might have been underpowered. Some centres had overlapping enrolment periods, raising the possibility of patient overlap, and studies reporting only adjusted estimates without arm-level event counts could not be pooled by the Mantel–Haenszel method. Randomized controlled trials with standardised outcome definitions, including cohorts using pulsed field ablation, are warranted to confirm our findings.

## Conclusion

In this meta-analysis, combined CA + LAAC was associated with a significantly higher rate of arrhythmia recurrence during follow-up than CA-only, a numerically lower but non-significant rate of systemic thromboembolism, and a comparable rate of periprocedural pericardial effusion requiring drainage. The higher recurrence rate is a clinically relevant signal that may reflect procedural factors such as limited substrate modification in a one-stop setting as well as differences in the underlying patient population, and it identifies the ablation strategy of the combined procedure as a key target for optimisation. The thromboembolic benefit appears concentrated in higher-risk patients. Combined CA + LAAC thus represents a feasible one-stop strategy whose net clinical value hinges on careful, risk-based patient selection and an adequate ablation strategy, and which warrants confirmation in adequately powered randomised trials.

## Supplementary Information

Below is the link to the electronic supplementary material.

Supplementary file1 (PNG 17 KB)

Supplementary file2 (PNG 15 KB)

Supplementary file3 (PNG 80 KB)

Supplementary file4 (PNG 15 KB)

Supplementary file5 (PNG 15 KB)

Supplementary file6 (PNG 60 KB)

Supplementary file7 (PNG 14 KB)

Supplementary file8 (PNG 15 KB)

Supplementary file9 (PNG 67 KB)

Supplementary file10 (PNG 15 KB)

Supplementary file11 (PNG 61 KB)

Supplementary file12 (PNG 14 KB)

Supplementary file13 (DOCX 16.8 KB)

Supplementary file14 (DOCX 14 KB)

## Data Availability

All data analysed in this study were extracted from previously published studies, which are cited in the manuscript. All data supporting the findings of this meta-analysis are available within the article and its supplementary information files. No new primary patient-level data were generated. Further details are available from the corresponding author on reasonable request.
