# Supplementary material for: Efficacy and safety of concomitant catheter ablation and left atrial appendage closure compared to catheter ablation only – a meta-analysis
Source: J Interv Card Electrophysiol. 2026 Aug 17;69(9):3013–23. doi: 10.1007/s10840-026-02418-3 (PMC13769750; doi:10.1007/s10840-026-02418-3)
Supplement: Supplementary file 1 [file 10840_2026_2418_MOESM13_ESM.docx]

| Supplemental Table 1. Timing, follow-up, arrhythmia recurrence definition, blanking period, monitoring, and antiarrhythmic drug protocol of included studies | | | | | | |
| --- | --- | --- | --- | --- | --- | --- |
| Study | Timing (LAAC/PVI) | Follow-up duration | Arrhythmia recurrence definition | Blanking period | Monitoring method | AAD protocol |
| Wang et al. 2022 | Concomitant | 12 months | No formal recurrence definition | N/A | N/A | N/A |
| Yang et al. 2021 | Concomitant  LAAC after PVI | 12 months | Atrial tachyarrhythmia >30 s | 3 months | 24-h monitor at 3, 6, and 12 months. | 3 months postoperatively for all patients |
| Pasupula et al. 2023 | Concomitant | 30-day readmission only | N/A | N/A | N/A | N/A |
| Romanov et al. 2015 | Concomitant  LAAC after PVI | 24 months | Atrial tachyarrhythmia >30 s | 3 months | Continuous ILR plus 24-h Holter at 3, 6, 9, 12, 18, and 24 months | 6 weeks postoperatively for all patients |
| Zhu et al. 2020 | Concomitant  LAAC after PVI | Mean ~ 12 months | Atrial tachyarrhythmia >30 s | 3 months | N/A | N/A |
| Pelissero et al. 2017 | Concomitant  LAAC before PVI | Mean ~ 12 months | No formal recurrence definition | 3 months | ILR in most patients; otherwise 24-h Holter at each visit. | No AADs prescribed at discharge |
| Mo et al. 2020 | Concomitant  LAAC after PVI | Mean ~ 24 months | No formal recurrence definition | 3 months | ECG or 24-h Holter at each visit (3, 6, 12 months, then every 6 months) | N/A |
| Yang et al. 2022 | Concomitant  LAAC after PVI | 3 months | No formal recurrence definition | 3 months | 24-h Holter at 1 and 3 months | 3 months postoperatively for all patients |
| Ding et al. 2024 | Concomitant  LAAC after PVI | Mean ~ 18 months | Atrial tachyarrhythmia >30 s | N/A | 24-h monitor plus portable ECG device at 3, 6, and 12 months. | N/A |
| Tam et al. 2025 | Concomitant  LAAC after PVI | 3 months | No formal recurrence definition | N/A | N/A | N/A |
| Ren et al. 2020 | Concomitant  LAAC after PVI | Median ~ 24 months | Atrial tachyarrhythmia >30 s | 3 months | 24-h or 7-day ECG monitor | 3 months postoperatively for all patients |
| Lv et al. 2024 | Concomitant  LAAC after PVI | 12 months | Atrial tachyarrhythmia >30 s | 3 months | 24-h ambulatory ECG at 3 and 12 months. | 3 months postoperatively for all patients |
| Yu et al. 2026 | Concomitant  LAAC before PVI | 48 months | Atrial tachyarrhythmia >30 s | 2 months | 12-lead ECG at every clinical visit; 24-h Holter at 6, 12, and 24 months. | N/A |
| Saliba et al. 2025 | Concomitant | 36 months | No formal recurrence definition | 3 months | N/A | N/A |
| LAAC, left atrial appendage closure; PVI, pulmonary vein isolation; AAD, anti-arrhythmic drug; N/A, not applicable/not available; ILR, implantable loop-recorder; ecg, electrocardiogram | | | | | | |
