# Supplementary material for: Efficacy and safety of concomitant catheter ablation and left atrial appendage closure compared to catheter ablation only – a meta-analysis
Source: J Interv Card Electrophysiol. 2026 Aug 17;69(9):3013–23. doi: 10.1007/s10840-026-02418-3 (PMC13769750; doi:10.1007/s10840-026-02418-3)
Supplement: Supplementary file 2 [file 10840_2026_2418_MOESM14_ESM.docx]

Search strategy

("left atrial appendage closure" OR "left atrial appendage occlusion" OR "LAA closure" OR "LAA occlusion" OR "LAAC" OR "LAAO" OR "Watchman" OR "Amulet" OR "transcatheter left atrial appendage" OR "percutaneous left atrial appendage")

AND

("pulmonary vein isolation" OR "pulmonary vein ablation" OR "catheter ablation" OR "cryoablation" OR "cryoballoon" OR "pulsed field ablation" OR "radiofrequency ablation" OR "PVI" OR "PFA" OR "atrial fibrillation ablation")

AND

("atrial fibrillation" OR "AF" OR "AFib")

NOT

("surgical" OR "thoracoscopic" OR "epicardial" OR "ligation" OR "stapler" OR "AtriClip" OR "LARIAT" OR "thoracotomy" OR "mini-thoracotomy")
